# Effectiveness and cost effectiveness of guided online treatment for patients with major depressive disorder on a waiting list for psychotherapy: study protocol of a randomized controlled trial

**DOI:** 10.1186/1745-6215-14-412

**Published:** 2013-12-01

**Authors:** Robin Maria Francisca Kenter, Annemieke van Straten, Sabine Heleen Hobbel, Filip Smit, Judith Bosmans, Aartjan Beekman, Pim Cuijpers

**Affiliations:** 1 Faculty of Psychology and Education, Department of Clinical Psychology, VU University Amsterdam, Van der Boechorststraat 1, 1081 BT Amsterdam, The Netherlands; 2 EMGO Institute for Health Care and Research, VU University Medical Centre, Van der Boechorststraat 1, 1081 BT Amsterdam, The Netherlands; 3 Trimbos Institute, Postbus 725, 3500 AS Utrecht, The Netherlands; 4 Department of Epidemiology and Biostatistics, VU University Medical Centre, Postbus 725, 3500 AS Utrecht, The Netherlands; 5 Department of Health Sciences, VU University Amsterdam and EMGO institute for Health Care, De Boelelaan 1085, 1081 HV Amsterdam, The Netherlands; 6 Research, VU University Medical Centre, De Boelelaan 1085, 1081 HV Amsterdam, The Netherlands; 7 Department of Psychiatry, VU University Medical Centre, De Boelelaan 1085, 1081 HV Amsterdam, The Netherlands

**Keywords:** Depressive disorders, Internet-based treatment, Online guided self-help, Cost effectiveness, Outpatients, Specialized mental healthcare

## Abstract

**Background:**

Depressive disorders are highly prevalent and result in negative consequences for both patients and society. It is therefore important that these disorders are treated adequately. However, due to increased demand for mental healthcare and subsequent increased costs, it would be desirable to reduce costs associated with major depressive disorder while maintaining or improving the quality of care within the healthcare system. Introducing evidence-based online self-help interventions in mental healthcare might be the way to maintain clinical effects while minimizing costs by reducing the number of face-to-face sessions. This study aims to evaluate the clinical and economical effects of a guided online self-help intervention when offered to patients with major depressive disorder on a waiting list for psychotherapy in specialized mental health centers (MHCs).

**Methods:**

Patients at mental health centers identified with a *Diagnostic and Statistical Manual of Mental Disorders*, fourth edition (DSM-IV) diagnosis of major depression who are awaiting face-to-face treatment are studied in a randomized controlled trial. During this waiting list period, patients are randomized and either (1) receive an internet-based guided self-help treatment or (2) receive a self-help book. The 5-week internet-based guided self-help intervention and the self-help booklet are based on problem solving treatment. After the intervention, patients are allowed to start regular face-to-face treatment at MHCs. Costs and effects are measured at baseline, after the intervention at 6 to 8 weeks, 6 months and 12 months. The primary outcome measure is symptoms of depression. Secondary outcome measures are diagnosis of depression, number of face-to-face sessions, absence of work and healthcare uptake in general. Additional outcome measures are anxiety, insomnia, quality of life and mastery.

**Discussion:**

This study evaluates the effectiveness and cost effectiveness of internet-based guided self-help in patients at specialized mental health centers. The aim is to demonstrate whether the introduction of internet-based self-help interventions in regular mental healthcare for depressive disorders can maintain clinical effects and reduce costs. Strengths and limitations of this study are discussed.

**Trial registration:**

Netherlands Trial Register NTR2824

## Background

Depressive disorders are highly prevalent [1-3], and are associated with high costs in the professional, social, personal and financial realms [1,3,4]. Those suffering from depression are more often absent from work, and have higher levels of healthcare consumption compared to healthy individuals [2]. It is desirable from a clinical point of view to reduce the burden of depression, as it is from a societal point of view to reduce the economic costs related to increased healthcare uptake, and reduced work productivity. In addition, given the increased demand for mental healthcare and limitation of recourses, it is important for mental health centers to optimize the efficient and effective use of resources.

In The Netherlands, a person with symptoms of depression who is seeking help is most likely to be seen first by a general practitioner (GP) [5], who acts as a gatekeeper for referral to specialized mental health services. After registration at a mental health service, patients usually have an assessment and are then assigned to a specific treatment. The time between registration and the first treatment session is normally at least 6 weeks. Long waiting lists caused by low workforce numbers are common. This time might be used to deploy internet-based self-help treatments [6,7], as previous studies have indicated that internet-based self-help therapies are clinically effective in diverse populations [8-11]. Internet-based treatments require less therapist time in comparison with standard face to face treatments and can therefore offer potential solutions as they are immediately accessible, less costly and put less strain on therapeutic resources. They might serve first step in a stepped delivery of care. This means that only those who do not respond adequately step up to a treatment of higher intensity, in this case the regular treatment in mental health centers. This stepped-care approach is suggested in several guidelines such as the Australian and National Institute for Health and Care Excellence (NICE) guidelines for depression, which recommend that patients receive the least burdensome treatment. Psychological treatments such as computerized cognitive behavioral therapy (cCBT) and individual guided self-help programs are recommended as low intensity treatments. After receiving such treatments, patients possibly need fewer or no face-to-face sessions.

With the current cuts in healthcare budgets, it would be desirable to reduce costs associated with major depressive disorder while maintaining or improving the quality of care. Introducing evidence-based internet-based self-help interventions in mental healthcare might be the way to speed up clinical recovery while minimizing costs by reducing the number of face-to-face sessions. However, internet-based guided self-help interventions are currently not offered as a first step to those waiting for specialized mental healthcare.

In the current study, we offer an internet-based guided self-help program to patients on a waitlist for psychological treatment. The self-help program is based on problem-solving therapy and uses self-examination therapy as a general framework [12]. This method has been found to be effective in several studies in the US [13], and for this study we used a guided self-help program that has been examined in two earlier trials, and has proved its effectiveness in reducing depressive symptoms in community samples [14,15].

### Aims and hypotheses

This study aims to establish the effectiveness of an internet-based guided self-help intervention for patients with major depressive disorder before face-to-face treatment in specialized mental healthcare in comparison to a control group who are on a waitlist for face-to-face treatment. Furthermore, we expect that offering internet-based treatment as a first step of care reduces costs as we predict the internet group to take up fewer regular treatment sessions.

## Methods

### Study design

The study is a randomized controlled trial with an economic evaluation. We are currently enrolling 248 patients over 2 large mental health centers (MHCs) in 10 different locations. We will recruit patients directly after registration for regular face-to-face mental health services who need to wait for at least 6 weeks before their first treatment session. They will be randomized to either an internet-based guided self-help intervention or the regular waitlist before the first treatment session. People on the waitlist will receive a self-help book without additional guidance in order to motivate them to participate in the randomize, controlled trial (RCT). Both groups are allowed to receive regular face-to-face (FTF) therapy at the MHCs after the waitlist period. Participants in both groups complete assessment at baseline, at intervention or waiting list (WL) completion (6 to 8 weeks), at a 6-month follow-up, and at 12 months. The protocol of this study has been approved by the Medical Ethics Committee of the VU University Medical Center (registration number 2011/223).

### Inclusion and exclusion criteria

Eligible participants are adults, aged 18 or over, registering for regular treatment at one of the participating MHCs who meet the criteria for a *Diagnostic and Statistical Manual of Mental Disorders*, fourth edition (DSM-IV) diagnosis of major depression as measured with the Clinical International Diagnostic Interview (CIDI) by a trained research assistant, have access to the internet, and adequate proficiency in Dutch. Exclusion criteria are starting or changing type of dosage of antidepressant medication, the presence of a bipolar or psychotic disorder, and an increased risk of suicide. Comorbid disorders other than bipolar or psychotic disorders are allowed.

### Recruitment

Participants will be recruited while registering at the participating MHCs. In routine care patients are briefly scanned by the MHCs (according to MHCs protocol to screen out patients who are in crisis and need immediate treatment), and for the purpose of this study, patients with mood problems who are eligible for an intake assessment are asked by the MHC to share their contact details with the researchers. Those who are willing to do so, will then be called by a member of the research team who further explains the aim of this study and performs an additional check of the inclusion and exclusion criteria. If eligible, patients receive a study brochure and an informed consent form. Patients will only be included in the study if they meet all the criteria, and sign the informed consent form. In order to confirm the diagnoses Major Depressive Disorder trained interviewers conduct a clinical interview (CIDI).

### Sample size

The trial is powered to detect an effect size of d ≥0.40 [10,16] as statistically significant in a two-tailed test with α = 0.05 and power of (1 - β =) 0.80 with N = 99 per condition. To compensate for loss of follow-up of 20%, the trial requires starting with 99/0.8 = 124 participants per condition at baseline. The dropout rate of 20% is conservative, but from a power perspective a cautious approach. As this trial has two conditions, a total of N = 248 will be necessary for the complete trial.

### Mental health centers characteristics

Two MHCs will be participating in this research. They each offer services at a number of different locations. In this trial, a total of ten locations will participate. These centers are chosen for pragmatic purposes, as they have a high number of patient enrolment, and have participated in prior research of the VU University. In general, patients are referred by their general practitioner to the mental health centers where the patients is screened and placed on a waiting list. Within 6 weeks an initial meeting with a therapist takes place in which the patient’s needs and preferences regarding to treatment are determined. At these participating MHCs it normally takes between 7 to 16 weeks for patients to have a first treatment session, depending on therapist workload, treatment modality and other factors. Treatment in both centers can consist of psychological therapies such as CBT, sometimes in combination with medication. The researchers are not involved in the face-to-face treatment, and neither in the decision-making process. However, prior to the start of this study all therapists at the participating MHCs will be informed about the goals of this study and the internet-based intervention, they will attend a Q&A session with the first author and receive the self-help book containing the intervention. Prior to the start of a patients’ face-to-face treatment at the MHC the therapists will be informed by the aforementioned note in electronic patient record and through email that their patients are participating in this study. One of the objectives of the internet-based intervention is that the subsequent face-to-face treatment can be adjusted to fewer sessions. The number and type of therapeutic sessions patients will receive is based on the needs of the patient, the judgment of the therapist, and protocol available at the MHC. The type and number of sessions will therefore vary per participant, and are outside the control of the researchers.

### Internet intervention

The internet intervention that will be used is called 'Taking Control’ (original title: 'Alles Onder Controle’). This intervention uses the self-examination treatment model developed by Bowman and colleagues [12,13], which is based on problem-solving therapy and uses self-examination therapy (SET) as a general framework. We translated it into Dutch, elaborated on it, and added information and exercises. We built a website for this intervention and developed a system for email support. This intervention has been described in more detail in several other studies [12-14].

In brief, this intervention is short, structured and manualized. It consists of five weekly sessions. Each session contains a structured homework assignment to be completed by the participant. The first session requires participants to consider what is important in their lives. Next to this, participants will make a list of current problems and worries in their lives. This first session helps participants to divide their problems into the following three categories: not important (if the problem is not related to the list of important things), important and unsolvable (for example, permanent loss of health or a loved one), or important and solvable. In the second, third and fourth session participants will be offered various coping skills related to each of the categories of problems; the main focus is placed on adopting a structured six-step approach when encountering important, solvable problems. This structured approach is divided in the following steps: identifying the current problem; finding possible solutions; selecting one solution; create a plan to solve the problem with this solution; execute the plan and evaluate the plan. The last week of the intervention is reserved for both the reflection on long-term goals and the development of a structure to achieve these goals. Participants can only move on to the next session once the exercise in the current session has been submitted and when feedback on this session has been released by the research team.

The participants are supported by a coach, who gives feedback to the homework assignments of the participants in brief, weekly emails. The total amount of time spent on each patient is about 1.5 h (estimate based on our earlier trials). The writing of these emails takes about 15 or 20 minutes per week, and will be performed by a coach. The coaches will be trained by the psychologists who have developed the intervention and also wrote the protocol for giving feedback to ensure the consistency and integrity. An independent psychologist will verify whether the coaches have followed the protocol sufficiently by reading a random selection of the feedback emails.

The feedback has two purposes. Firstly, coaches will help participants to become familiar with the presented techniques. The second purpose consists of motivating the participant to continue with the intervention. Feedback will be received by participants within 3 working days after a session has been completed and submitted. When participants pose content related questions to their coaches via the website, email or phone, their coaches will provide additional guidance after receiving the question. After 5 weeks of guidance, patients can continue to use the internet-based treatment but will not receive any feedback on their assignments.

### Control condition

To increase participation rates in the control group, this group receives an unguided self-help book posted to their home address. This control group will not receive any feedback from coaches, nor will it have the opportunity to pose questions. Earlier research shows that self-help without any form of guidance only results in a small effect on participants with increased levels of depressive symptoms [16].

Participants in both conditions will be referred for regular FTF treatment after registration at the MHC. FTF treatment might consist of additional waiting time; the duration of the waiting time is highly variable with a minimum of 7 weeks and a maximum of 16 weeks depending on the location of MHC. Variation fluctuates both between locations and over time within the centers, due to availability of therapists. In case the waiting time for any MHC falls below 8 weeks, new participants from this MHC will be temporarily excluded from participating in the research until the waiting time for new patients at this MHC exceeds 8 weeks again. This has been decided in order to not measure the effects of active treatment by the MHCs at the first post-intervention test at 8 weeks.

The researchers of this study do not influence the waiting time at the MHCs, nor will participation in this study influence the waiting time for the participants.

### Randomization and blinding

**Figure 1:**
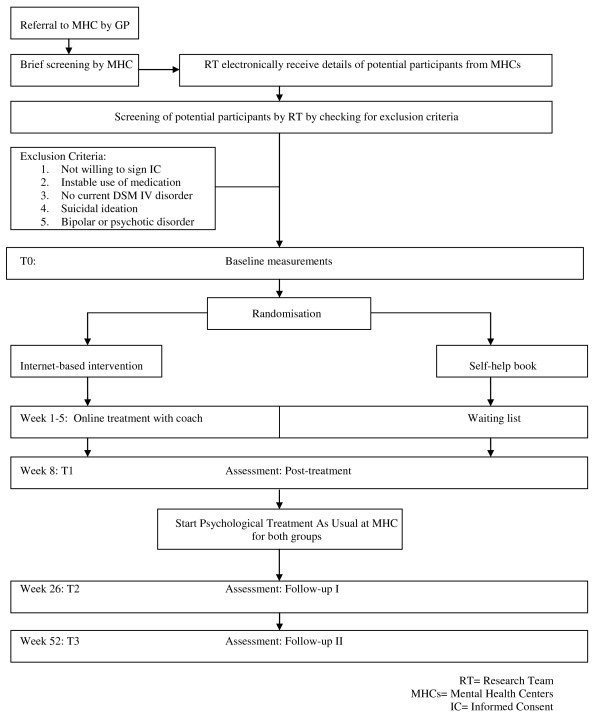
Study flow chart.

The random allocation sequence will be generated by an independent researcher in the program 'Random Allocation Software’, stratified by location using blocks of six, eight and ten. After each inclusion another independent researcher will allocate the patient to either the intervention or the control condition. Due to the nature of the intervention the treatment group allocation cannot be concealed from the participants, nor from the research team and assessor of outcomes. Participants are assigned to one of two conditions within 2 working days from their baseline assessment Figure 1.

### Assessments

This study will utilize both clinical and economical outcome measures (see Table 1). Assessments take place at baseline (before randomization), follow-up assessments are at post-intervention (6 to 8 weeks), and at 6 and 12 months after baseline.

**Table 1:** Outcome measures

**Questionnaires**	**Aim**	**T0 Baseline (pre-test)**	**T1 Post-test (8 weeks)**	**T2 Follow-up I (6 months)**	**T3 Follow-up II (12 months)**
Primary outcome					
CES-D	Symptoms of depression	X	X	X	X
Secondary outcomes					
CIDI (Section E)	Diagnosis of depression	X	X		
HADS (Anxiety section)	Symptoms of anxiety	X	X		
ISI	Level of insomnia	X	X		
EQ-5D	Quality of life	X	X	X	X
Resource use					
F2F	Number of face-to-face appointments in MHC				X
TiC-P	General health service uptake and productivity losses	X	X	X	X
Mediator					
Mastery scale	Mastery	X	X	X	X
Moderators					
Demographic factors	User characteristics	X			
NEO-FFI (sections N + C)	Neuroticism and conscientiousness traits	X			
CAGE	Alcohol consumption	X			
CEQ	Expectancy and treatment	X			
AOCEQ	Expectations of the internet intervention	X			
CSQ-8	Client satisfaction with treatment		X		
AOCSQ	Satisfaction with type of intervention		X		

*AOCEQ* 'Alles Onder Controle’ Expectation Questionnaire, *AOCSQ* 'Alles Onder Controle’ Satisfaction Questionnaire, *CAGE* 'Cutting down, Annoyance by criticism, Guilty feeling, and Eye-openers’, *CEQ* Credibility/Expectancy Questionnaire, *CES-D* Center for Epidemiological Studies Depression scale, *CIDI* Composite International Diagnostic Interview, *CSQ-8* Client Satisfactory Questionnaire-8, *EQ-5D* EuroQol 5-Dimensions, *F2F* face-to-face, *ISI* Insomnia Severity Index, *HADS* Hospital Anxiety Depression Scale, *MHC* mental health center, *NEO-FFI* NEO-Five Factor Inventory, *TiC-P* Trimbos and iMTA Questionnaire on Costs Associated with Psychiatric Illness.

### Outcome measures

Our primary outcome will be symptoms of depression. Secondary outcomes are DSM-IV diagnosis of depression, number of face-to-face sessions in MHC, costs (in terms of all healthcare use and work absenteeism), anxiety, mastery, insomnia, quality of life, and satisfaction. Plausible moderators measured in this study will be alcohol use, personality, demographic factors and client expectations.

Because the Composite International Diagnostic Interview (CIDI) is part of the questionnaires at baseline and at post-intervention assessment (Table 1), the baseline questionnaires and the 6 to 8 week assessment will be administered by phone. Follow-up assessments at 6 and 12 months will be administered online. All questionnaires will be in Dutch.

### Primary outcome

#### Symptoms of depression

Symptoms of depression will be registered by using the Center for Epidemiological Studies Depression scale (CES-D) [17]. This scale consists of 20 items, the total score ranges between 0 and 60; higher scores indicate higher levels of depression and a score of 16 and above indicates a clinical level of depression. This questionnaire has been tested in various populations and has been found valid and reliable [18]. A Dutch version of the CES-D has been validated for internet administration [19].

### Secondary outcomes

#### Diagnosis of depression

The CIDI will be used to assess whether the DSM-IV diagnosis of depression has been met. The CIDI has been developed by the World Health Organization to assess DSM-IV Axis-I diagnoses [20]. For the purposes of this study, only section E will be administered, which allows for screening for depressive disorders. The CIDI will be conducted by phone by a trained interviewer.

#### Symptoms of anxiety

The subscale Anxiety of the Hospital Anxiety and Depression Scale (HADS) will be used to measure symptoms of anxiety. The depression scale will not be utilized in this research because depression will be measured by both CIDI and CES-D. The Anxiety subscale consists of 7 items, which scores range from 0 to 21; higher scores indicate higher levels of anxiety [21]. The HADS has shown to be reliable in Dutch populations [21].

#### Symptoms of insomnia

The perceived level of insomnia will be measured by the Insomnia Severity Index (ISI) [22]. This questionnaire measures both the concerns associated with the perceived level of insomnia, as well as symptoms and consequences of insomnia. Each item is rated on a 0 to 4 scale; a higher score indicates more severe insomnia. ISI has been found to be internally consistent and reliable [22].

#### Quality of life

The EuroQol 5-Dimensions questionnaire (EQ-5D), which consists of five items, will be used to measure quality of life. It registers the level of perceived problems (no, some or extreme) in five domains (mobility, self-care, usual activities, pain/discomfort and anxiety/depression). A total of 486 distinct health states can be scored which are located between 0, which indicates worst health possible, and 1, which indicates perfect health [23].

### Resource use

#### Costs related to the intervention

Costs related to the intervention will be calculated based on the costs of running the intervention in both conditions. To calculate costs related to the internet intervention, the costs of running the website platform and the costs of the hours of coaching invested in the intervention will be taken into account. Additional costs in the control group are the publishing of the self-help books.

#### Costs related to mental healthcare

Costs related to mental healthcare will be measured as the reported number and type of sessions by the MHC. This information is sent to the first author 12 months after participants have registered at the MHCs. The direct costs of the sessions will be calculated based on the Dutch standard cost prices [24].

#### Costs from a societal perspective

Healthcare costs in general as well as productivity losses will be measured with the revised version of the Trimbos and iMTA Questionnaire on Costs Associated with Psychiatric Illness (TiC-P). Direct costs will be measured by investigating which contact with health professionals has occurred and which type of medication has been prescribed. Indirect costs will be measured by work absenteeism and reduced productivity. The baseline TiC-P measures the care consumption 4 weeks prior to the intervention. The follow-up assessments at 6 and 12 months use a 14 and 26 weeks recall period respectively. Previous research has shown that up until half a year later, patients can reliably recall their consumption of health services [25]. The TiC-P has been used previously in a population with depressive symptoms in The Netherlands [26].

### Mediator

#### Mediator

The amount of perceived control in a person’s life will be measured by the Pearlin Mastery Scale [27]. The scale consists of seven distinct items that are rated on a four-point scale. Higher scores indicate more perceived control; scores range from 7 to 35. The scale has good reliability [27].

### Moderators

#### Demographics

Demographic variables such as age, gender, parental nationality, family composition, family income and educational level will be screened for in a general questionnaire that measures user characteristics.

#### Alcohol use

The use of alcohol will be monitored using the four questions that make up the acronym CAGE: 'Cutting down, Annoyance by criticism, Guilty feeling, and Eye-openers’ [28]. The CAGE is a widely used concise screening tool for problematic alcohol consumption [29].

#### Personality

In order to measure the constructs neuroticism and conscientiousness, the NEO-Five Factor Inventory (NEO-FFI) will be administered [30]. Previous research has indicated that neuroticism often coincides with depression (for example, [31]). Those scoring higher on conscientiousness are expected to adhere better to the homework exercises and consequently benefit more from internet-based therapies. Only these two domains will be tested for to not exhaust participants more than necessary. A total of 12 questions on each of the domains will be answered on a 5-point Likert scale.

#### General expectancy

The Credibility/Expectancy Questionnaire of Devilly and Borkovec (CEQ) will be used to measure the expected change and credibility of proposed treatment [32]. It consists of six questions; four questions measure the 'thinking’ aspects about the treatment, two questions measure the 'feeling’ aspects of the questions. One question in both the 'feeling’ and 'thinking’ domain is rated from 0% to 100%; the remaining four questions are rated on a Likert-type scale from 1 to 9. Both factors have been found to have a high internal consistency and to have high test-retest reliability [32].

#### Expectancy with internet intervention

The 'Alles Onder Controle’ Expectancy Questionnaire (AOCEQ) has been designed for this research to ask what participants’ expectations are about the course. An open question investigates in which ways participants expect to gain benefits from this course. Three five-point Likert scale questions measure to which degree participants appreciate starting with the intervention immediately, that personalized feedback will be given in the internet group and to which degree they expect the course to help them feel less miserable.

#### General satisfaction with treatment

The satisfaction with the internet intervention will be measured by the Client Satisfactory Questionnaire-8 (CSQ-8), which consists of eight questions, each question is scored on a Likert-type scale from 1 to 4 [33]. The questionnaire addresses several elements that contribute to overall service satisfaction and is reported in a single dimension of overall satisfaction. A high internal consistency has been reported [33].

#### Satisfaction with the internet intervention

The 'Alles Onder Controle’ Satisfaction Questionnaire (AOCSQ) has been designed specifically to investigate to what degree participants are satisfied with this internet-based intervention. It includes questions on the number of sessions completed and, if applicable, the reasons for not finishing the course. The questionnaire further researches satisfaction with the separate elements of the intervention, such as the quality of the feedback, the clarity of the website and the appropriateness of the examples. Lastly, it explores whether participants were satisfied with the provided alternative to waiting, to which degree internet interventions are preferred over book interventions and to which degree feedback is preferred over non-feedback.

### Statistical analysis

The analyses will be conducted in agreement with the intention to treat (ITT) principle, as per the CONSORT statement [34]. Therefore missing endpoints will be imputed using state of the art imputation methods, as a reliable method for handling missing values [35]. Imputation allows for analyzing all participants in the condition to which they have been randomized, which contributes to guarantee the integrity of the randomization and restores loss of power due to dropout.

To answer the research questions we will first look at post-test differences between the two groups. We will use analysis of variance (ANOVA), with the baseline values and waiting time as covariates. Subgroup analyses will be performed for different characteristics. The difference in scores between the intervention group and the control group will also be expressed in effect sizes. We use Cohen’s *d* which is calculated by dividing the difference in mean scores of the two groups by their pooled standard deviation (Xexp-Xctrl/SDpooled). Effect sizes under 0.2 are considered to be small, those of 0.5 are moderate and effect sizes of 0.8 are considered to be large [36]. Furthermore, we will compare the rate of DSM-IV diagnosis of depression in both groups with logistic regression analysis. The clinical effects will also be calculated using reliable change [37]. The long-term outcomes will be analyzed with longitudinal analyses.

### Economic outcomes

The economic evaluation will be conducted from a societal perspective, therefore it will include not only the intervention costs and costs stemming from healthcare uptake (direct medical costs), but also the patients' out of pocket costs (direct non-medical costs) and costs stemming from productivity losses due to absenteeism and work cutback days (indirect non-medical costs). Costs will be based on the Dutch standard cost prices [24] and productivity losses will be valued using the friction costs method, as per the Dutch guideline for economic evaluation. Quality-adjusted life years (QALYs) will be calculated on the basis of the EQ-5D. Having calculated the costs and QALYs allows for a cost-utility analysis, which can be used to compare this intervention’s gains against those of other interventions for depressive disorders. A cost-effectiveness analysis can be carried out by dividing the difference in costs by the difference in effect, as is customary in the field of mental health. Bootstrapping will be used to ascertain the amount of uncertainty surrounding the incremental cost effectiveness ratio (ICER) estimates and graphically depicted on the ICER plane and in the acceptability curve.

## Discussion

This study will examine the effectiveness of offering an internet-based guided self-help intervention to patients before to start of face-to-face treatment in comparison with patients who have to wait for face-to-face treatment. We will also compare the uptake of regular treatment in both groups. An economic evaluation will determine whether a guided internet intervention followed by face-to-face treatment is economically more sensible compared to waiting for face-to-face treatment. A number of strengths and challenges have been identified by the researchers in this study.

### Strengths

Existing evidence shows that internet-based treatments are effective in treating depressive disorders in general populations [9,38]. This trial however, will shed new light on the question whether patients with major depressive disorder waiting for specialized care in MHCs can benefit from this type of internet-based guided self-help intervention. This population has, to the best of our knowledge, not previously been studied in this manner, while the relevance of conducting a study on people with major depressive disorder who receive an internet-based intervention as a first step of treatment is high for a number of reasons. As demonstrated previously, depression is a widely prevalent, invasive disorder, which affects the patients, their direct environments, and the society as a whole in multiple ways. Any measures which contribute to more people receiving better care for their depression in specialized mental healthcare against a lower costs will be welcomed by all stakeholders. Stepped care is suggested for treating depression in multiple guidelines, for example the NICE guidelines in the UK (2009) Although there is some supporting evidence, there are few RCTs to demonstrate the (cost) effectiveness of this program in specialized mental healthcare. Furthermore, considering the increase of online interventions to the standard treatment in more and more MHCs, this study will contribute valuable information on the effects of adding online treatment as a first step towards treating major depressive disorder.

An additional strength of this intervention is the online delivery. The online intervention allows participants not only to access the intervention any convenient hour, with the rise of portable internet there are hardly any limitations related to the location of a participant. In addition, offering guided online treatment during the time otherwise lost to waiting may result in benefits for the patient as well as for the MHCs in terms of patient satisfaction and clinical effectiveness.

A final strength of this study is the possibility of applicability. If the study indicates that it is economically and clinically beneficial to deliver internet interventions to those with a major depressive disorder as part of their treatment, the intervention could easily be integrated as a standard component in the treatment of depression, as only short training is necessary to become a successful coach for this intervention. This intervention could be applied widely in case the intervention is beneficial for treating people with major depressive disorder.

### Limitations

One of the possible challenges of this study concerns the general attrition from and non-adherence to internet interventions. Internet treatments require a degree of motivation of the participant. This study includes depressed patients who, by definition, have impaired motivation and lack of energy that may make it more difficult for patients adhere to the intervention. To prevent dropouts and maximize the uptake rates the participants will receive emails and phone calls stating the importance adhering to the intervention.

Another challenge for the trial could be the prevailing attitude of psychologists at several MHCs who fear redundancy due to internet interventions. However, when MHCs would start treating patients online as a first step towards better health, more patients could be reached as online treatment seems to be less time consuming for health professionals compared to face-to-face treatment.

A further challenge is the extent the therapists take into account that patients might already have acquired skills and knowledge due to the internet intervention, so that there might be no need to start patients a fixed number of face-to-face sessions as prescribed by the standard treatment protocol. We aim that only those patients who require additional treatment due to a more complex psychological problem will be seen face-to-face. Therefore, if internet interventions are proven to be more clinically effective, it is feasible that psychologists at MHCs will be able to treat more patients in a better manner because of the implementation of internet interventions.

In summary, existing internet-based guided self-help treatments focus mainly on depression in the general population. This trial, however, is to the best of our knowledge the first effectiveness study of an internet-based guided self-help intervention for major depressive disorder in specialized mental healthcare that also focuses on reduction of face-to-face sessions and costs in general. The findings of this study will contribute to the body of knowledge on the additional value of internet-based treatments for depression. And if the low intensity internet intervention shows to be (cost) effective, it might serve as a first step towards the treatment of major depressive disorder.

## Trial status

The status of the trial is ongoing recruitment.

## Competing interests

The authors declare that they have no competing interests.

## Authors’ contributions

PC, AvS and JB obtained funding for this study. PC, AvS, JB, and FS contributed to the design of the study. PC and AvS created the intervention. RK coordinates the recruitment of patients and data collection. AB, PC and AvS are responsible for the overall supervision. RK and SHH wrote the manuscript. All authors read, contributed towards and approved of the final manuscript.
